# Prediction of adverse cardiac events in emergency department patients with chest pain using machine learning for variable selection

**DOI:** 10.1186/1472-6947-14-75

**Published:** 2014-08-23

**Authors:** Nan Liu, Zhi Xiong Koh, Junyang Goh, Zhiping Lin, Benjamin Haaland, Boon Ping Ting, Marcus Eng Hock Ong

**Affiliations:** 1 Department of Emergency Medicine, Singapore General Hospital, Outram Road, Singapore 169608, Singapore; 2 Changi General Hospital, Singapore 529889, Singapore; 3 School of Electrical and Electronic Engineering, Nanyang Technological University, Singapore 639798, Singapore; 4 Duke-NUS Graduate Medical School, Singapore 169857, Singapore

**Keywords:** Triage, Major adverse cardiac events, Scoring system, Random forest

## Abstract

**Background:**

The key aim of triage in chest pain patients is to identify those with high risk of adverse cardiac events as they require intensive monitoring and early intervention. In this study, we aim to discover the most relevant variables for risk prediction of major adverse cardiac events (MACE) using clinical signs and heart rate variability.

**Methods:**

A total of 702 chest pain patients at the Emergency Department (ED) of a tertiary hospital in Singapore were included in this study. The recruited patients were at least 30 years of age and who presented to the ED with a primary complaint of non-traumatic chest pain. The primary outcome was a composite of MACE such as death and cardiac arrest within 72 h of arrival at the ED. For each patient, eight clinical signs such as blood pressure and temperature were measured, and a 5-min ECG was recorded to derive heart rate variability parameters. A random forest-based novel method was developed to select the most relevant variables. A geometric distance-based machine learning scoring system was then implemented to derive a risk score from 0 to 100.

**Results:**

Out of 702 patients, 29 (4.1%) met the primary outcome. We selected the 3 most relevant variables for predicting MACE, which were systolic blood pressure, the mean RR interval and the mean instantaneous heart rate. The scoring system with these 3 variables produced an area under the curve (AUC) of 0.812, and a cutoff score of 43 gave a sensitivity of 82.8% and specificity of 63.4%, while the scoring system with all the 23 variables had an AUC of 0.736, and a cutoff score of 49 gave a sensitivity of 72.4% and specificity of 63.0%. Conventional thrombolysis in myocardial infarction score and the modified early warning score achieved AUC values of 0.637 and 0.622, respectively.

**Conclusions:**

It is observed that a few predictors outperformed the whole set of variables in predicting MACE within 72 h. We conclude that more predictors do not necessarily guarantee better prediction results. Furthermore, machine learning-based variable selection seems promising in discovering a few relevant and significant measures as predictors.

## Background

Chest pain is one of the leading causes of visits to the emergency department (ED) [1]. Patients with chest pain present a wide range of risk for death and adverse cardiac events [2]. Of great concern is the risk of cardiac arrest that accounts for the majority of early deaths in patients with acute myocardial infarction (AMI) and other adverse cardiac events [3]. Significant hospital resources are dedicated to these high risk patients. Therefore, accurate risk stratification of chest pain patients for the prediction of major adverse cardiac events (MACE) could play an essential role in supporting clinical decisions that allow timely intervention for preventable and treatable complications. It could also allow management of low-risk patients without unnecessary admissions, investigations and monitoring, hence reducing the strain on limited ED resources. Such a risk stratification tool would be useful for chest pain patients presenting to the ED [3].

Various systems exist for stratifying the risk of acute coronary syndromes (ACS) [4-6]. The thrombolysis in myocardial infarction (TIMI) [5] score and the Global Registry of Acute Coronary Events (GRACE) [6] score were developed to predict the risk of death, reinfarction and revascularization. TIMI and GRACE scores have been validated on an unselected population of chest pain patients at the ED for predicting adverse events. However, both risk scores are often not applicable at the first presentation [7] as not all the variables are measured routinely in the ED. The modified early warning score (MEWS) is another popular tool in Commonwealth countries to identify patients at risk of deterioration [8,9]. Its process of risk evaluation involves assessment of vital signs and accurate prediction requires some prior training [10]. Furthermore, the above mentioned scoring systems use predefined predictive variables, prohibiting them from adopting new variables that are clinically significant for risk stratification. Several limitations have been reported in current risk scores for prediction of cardiovascular complications [11,12].

Most scoring systems use clinical vital signs such as heart rate, respiratory rate, blood pressure, temperature and pulse oximetry for risk stratification model derivation [13], but these physiological measures might not correlate well with short- or long-term clinical outcomes [14,15]. Furthermore, conventional statistical scoring systems are usually not readily adaptable to new variables [16]. Motivated by the flexibility of machine learning (ML) techniques, we have previously developed an intelligent scoring system [17] for predicting acute cardiac complications and discovered that rapid non-invasive bedside heart rate variability (HRV) combined with clinical signs showed improved prediction performance when compared with the MEWS [10]. However, redundant information likely exists within these predictive variables as not all the variables may contribute to the prediction [18]. Machine learning-based variable selection has been widely used in bioinformatics [19] but received little attention in medical research where traditional statistical methods play a dominant role [20]. In this study, we aim to discover the most relevant variables from HRV and clinical signs for the prediction of MACE using machine learning. We will compare the aforementioned ML score [17] using the selected variables with TIMI and MEWS scores by conducting the receiver operating characteristic (ROC) analysis for performance evaluation [21].

## Methods

### Study design

This was a prospective observational study on a convenience sample of 702 patients presenting to the ED of SGH, a tertiary hospital in Singapore, serving more than 135,000 patients in the emergency care setting annually. Patients who were at least 30 years of age and with undifferentiated non-traumatic chest pain were included in the study. Monitoring was done with an ECG sensor (Vernier Software & Technology, Portland, OR) and a data acquisition device (NI USB-6215, National Instruments, Austin, TX) over an uninterrupted recording period of 5 minutes. Patients were excluded if their ECG recordings contained sustained arrhythmias or large segments of noise or artifact. Patients who were transferred to another hospital or discharged against medical advice within 72 h of arrival at the ED were also excluded. Following the guidelines by the Task Force of the European Society of Cardiology and the North American Society of Pacing and Electrophysiology [22], a total of 15 HRV parameters shown in Table 1 were computed. During recording of patients’ ECG, their clinical signs were measured by attending nurses or physicians. Eight clinical signs were used, including systolic blood pressure (BP), diastolic BP, respiratory rate, heart rate, Glasgow coma scale (GCS), temperature, pain score (1–10), and oxygen saturation (SpO_2_). Demographic data such as age, race, gender, and medical history were also retrieved from the ED charts.

**Table 1:** List of HRV parameters and their definitions

**HRV parameter (unit)**	**Definition**
aRR (s)	Average width of the RR interval
STD (s)	Standard deviation of all RR intervals
avHR (beats/minute)	Average of the instantaneous heart rate (HR)
sdHR (beats/minute)	Standard deviation of the instantaneous HR
RMSSD (s)	Root mean square of differences between adjacent RR intervals
NN50 (count)	Number of consecutive RR intervals differing by more than 50 ms
pNN50 (%)	Number and percentage of consecutive RR intervals differing by more than 50 ms
Triangular index	Total number of all RR intervals divided by the height of the histogram of intervals
TINN	Baseline width of a triangle fit into the RR interval histogram using a least squares
LF power (ms^2^)	Power in low frequency range 0.04-0.15 Hz
HF power (ms^2^)	Power in high frequency range 0.15-0.40 Hz
Total power (ms^2^)	Total power estimated from RR intervals
LF norm (n.u.)	LF power in normalized units: LF/(Total power-VLF) × 100
HF norm (n.u.)	HF power in normalized units: HF/(Total power-VLF) × 100
LF/HF	Ratio of LF power to HF power

VLF: Very low frequency power in range ≤ 0.04 Hz.

Patients were followed up to discharge from hospital or in-hospital death. The primary outcome was defined as a composite of four major adverse cardiac events (MACE) within 72 h of patient’s arrival at the ED. MACE in this study included death, cardiac arrest, sustained ventricular tachycardia (VT), and hypotension requiring inotropes or intra-aortic balloon pump (IABP) insertion. The outcomes were retrieved from the ED charts and outcome decisions were made by physicians.

Ethics approval was obtained from the Singapore Health Services (SingHealth) Centralized Institutional Review Board (CIRB Ref: 2009/871/C) with a waiver of patient consent. This study was conducted from March 2010 to April 2012 at the Emergency Department (ED) of Singapore General Hospital (SGH).

### Predictive variable selection

Variable selection in statistics and machine learning is a process of determining a subset of relevant variables for model construction. Irrelevant variables usually do not contribute to model building and may even degrade the prediction performance. Automatic variable selection methods have been widely adopted in clinical studies such as for predicting acute myocardial infarction mortality [23].

We have previously studied the use of combined HRV parameters and clinical signs for patient outcome prediction [17,24] where each variable was assumed to equally contribute to model building. These studies could be refined by means of variable selection such that predictive variables are individually evaluated for their correlations to the primary outcome. Refinement of the model required a novel variable selection method as the distribution of our data was imbalanced; relatively few patients met the primary outcome (29 out of 702). Data imbalance compromises the performance of most machine learning algorithms [25].

In this study, we proposed a novel variable selection framework based on ensemble learning, in which random forests (RF) [26] was chosen as the independent variable selector for creating the decision ensemble, where the number of trees was 500. RF is an ensemble learning method for classification and regression. It combines many binary decision trees built using several bootstrapped learning samples and choosing randomly at each node a subset of explanatory variables [27]. The RF approach has been shown to be effective in variable selection [27,28].

**Figure 1:**
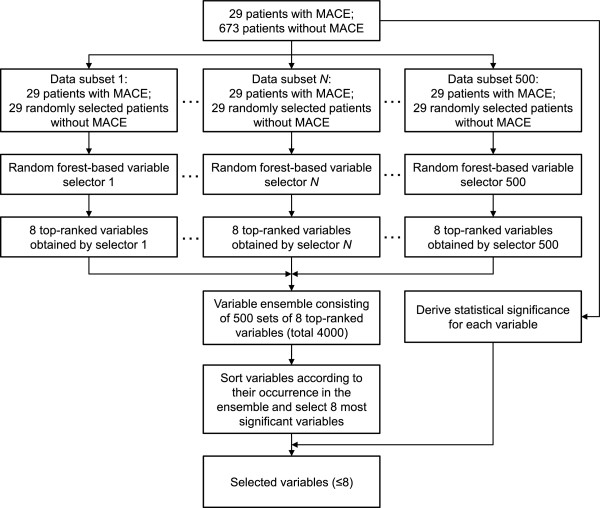
**Variable selection algorithm.** This algorithm creates 500 data subsets for subsequent analysis. Each subset combines 29 patients with MACE and 29 randomly selected patients without MACE. Then, the algorithm runs random forest on each subset to pick 8 top-ranked variables. Having 500 sets of top-ranked variables, the algorithm sorts them according to their corresponding occurrence in the ensemble and chooses 8 variables with the highest appearance. The selection is refined by means of the statistical significance of each individual variable.

Our proposed variable selection framework is elaborated as follows. Firstly, 29 out of 673 patients (without MACE) were randomly selected and combined with all 29 patients (with MACE) to construct a new subset, on which RF was used to pick eight top-ranked variables. Secondly, the above random sampling process was repeated 500 times to create an ensemble of top-ranked variables. Thirdly, variables in the ensemble were accumulated and sorted according to their corresponding occurrences. As a result, a total of 500 individual models were created by RF, with each picking eight variables to form an ensemble of predictors. In this particular study, eight top-ranked variables were determined as potential predictors of the primary outcome. To optimize the selected variables for future validation, 10-fold cross-validation was implemented to avoid over-training during model construction. Lastly, the statistical significance of each variable was measured. If any one of the eight selected variables was not significant in terms of *p*-value, it was excluded. Figure 1 depicts the variable selection method.

The classification and regression training package [29] in R programming language was used to implement RF for variable selection. Data were imported into R from a CSV file where all variables were in continuous format and the primary outcome was in categorical format.

### Risk score prediction

**Figure 2:**
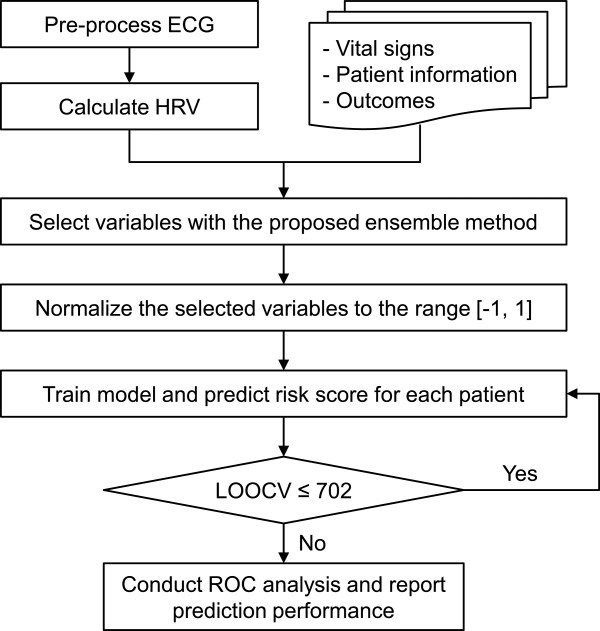
Flowchart of the machine learning-based risk scoring method.

Variable selection was the process of choosing a set of variables for the subsequent risk prediction. In this study, a machine learning (ML) based intelligent scoring system [17] (subsequently referred to here as the ML score) was implemented to build prediction models. The ML method examines geometric distances in Euclidean space between a testing sample and the training samples and produces a score on the possibility that the outcome of the testing sample approximates to the primary outcome. The ML method is illustrated in Figure 2 and is briefly described as follows: Firstly, the selected variables were converted into interval [-1, 1] with min-max normalization [30]. Secondly, cluster centers for both positive samples (patients with MACE) and negative samples (patients without MACE) were calculated based on Euclidean distance, and an initial score for a testing sample was derived by measuring distances between the testing sample and two cluster centers. Lastly, the support vector machine (SVM) [31] was implemented to fine-tune the risk score. Details of the ML method are described in [17].

In this study only a set of the selected variables was fed into the learning model for risk prediction, which was different from our previous ML method [17] that utilized all variables (15 HRV parameters and eight clinical signs) as the input. The output risk score ranges from 0 to 100 with 0 indicating no risk and 100 indicating the highest risk of MACE within 72 h. To compare the ML score with existing clinical scores, TIMI and MEWS scores were also derived for each patient.

### Statistical analysis

Continuous variables were presented as means (standard deviation) or medians (interquartile range) and were analyzed using the Mann–Whitney test. SPSS version 17.0 (SPSS Inc., Chicago, IL), MATLAB R2009a (Mathworks, Natick, MA), and R version 2.15.1 (R Foundation, Vienna, Austria) were employed for data analysis.

Performance evaluation was carried out with the leave-one-out cross-validation (LOOCV) strategy to get unbiased estimation of the model performance. In this study with 702 samples, 702 iterations were required for performance evaluation. In iteration, one sample was used as the testing sample while the remaining 701 samples were used for training. The risk score prediction process was repeated 702 times so that each sample was tested individually. Risk scores were then obtained for the entire dataset and a threshold was derived to report sensitivity and specificity.

A package developed in MATLAB was used to analyze ECG for HRV, calculate risk scores, and report prediction performance measures. To further evaluate differences in discrimination between models, pair-wise AUC comparisons using bootstrap method were performed with a ROC comparison package [32]. Statistical significance was set at *p*-value <0.05.

## Results and discussion

### Results

A total of 702 patients were recruited for the study; 29 of them met the primary outcome (MACE within 72 h). Characteristics of recruited patients are presented in Table 2. Males made up 66% of the study cohort. Chinese, Malay and Indian were the top three race groups, which matched the demographics of Singapore. Medical history of diabetes, stroke, chronic renal failure, congestive heart failure and myocardial infarction were more frequently observed in patients with MACE within 72 h than in patients without MACE. Table 3 shows the outcomes of patients with MACE within 72 h of arrival at the ED. It is observed that patients who had MACE within 72 h were more likely to develop one or more severe complications.

**Table 2:** Characteristics of the recruited patients

	**No MACE within 72 h**	**MACE within 72 h**
	**(N = 673)**	**(N = 29)**
Mean age (SD)	60.6 (13.0)	61.0 (11.6)
Male gender	445 (66.1)	18 (62.1)
Race		
Chinese	434 (64.5)	20 (69.0)
Malay	132 (19.6)	6 (20.7)
Indian	89 (13.2)	2 (6.9)
Others	18 (2.7)	1 (3.4)
Medical history		
Ischemic heart disease	292 (43.4)	10 (34.5)
Diabetes	241 (35.8)	13 (44.8)
Hypertension	432 (64.2)	17 (58.6)
Dyslipidemia	403 (59.9)	13 (44.8)
Stroke	50 (7.4)	3 (10.3)
Cancer	28 (4.2)	1 (3.4)
Chronic renal failure	79 (11.7)	9 (31.0)
Congestive heart failure	37 (5.5)	3 (10.3)
Respiratory disease	18 (2.7)	0 (0.0)
Myocardial infarction	96 (14.3)	6 (20.7)
PCI	149 (22.1)	4 (13.8)
CABG	62 (9.2)	1 (3.4)

Data are shown as numbers (%) unless otherwise stated.

MACE: major adverse cardiac events; PCI: percutaneous coronary intervention; CABG: coronary artery bypass graft.

**Table 3:** Outcomes of patients with MACE within 72 h of arrival at the ED

**Event**	**Number of patients (%)**
One or more severe complications	29 (4.1)
Death	9 (1.3)
Cardiac arrest	10 (1.4)
Sustained ventricular tachycardia	8 (1.1)
Hypotension requiring inotropes or IABP insertion	16 (2.3)

MACE: major adverse cardiac events; IABP: intra-aortic balloon pump.

**Figure 3:**
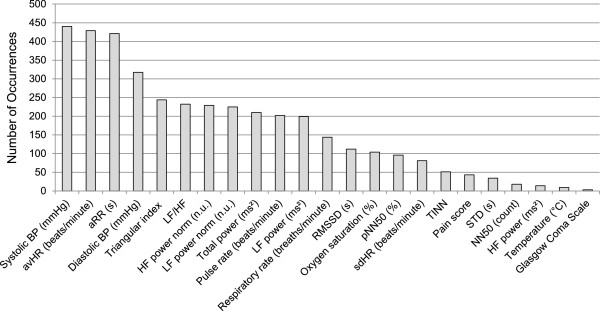
**Individual variables and their corresponding occurrences in the ensemble for variable selection.** The occurrence indicates the total number of appearance for a single variable in 500 random forest-based variable selectors. Therefore, the upper bound of the occurrence is 500.

Figure 3 presents the 23 individual variables and their corresponding occurrences in the ensemble for variable selection. Systolic BP (SBP), avHR, aRR, diastolic BP (DBP), triangular index (TI), LF/HF, HF power norm, and LF power norm were the eight top-ranked predictors associated with the primary outcome. These selected variables were fed into the intelligent scoring system [17] for risk prediction. Twenty-three variables consisting of 15 HRV parameters and 8 clinical signs are shown in Table 4. A predictor is considered significant if it has a *p*-value of <0.05. Temperature, oxygen saturation and pain score (clinical signs), and STD, sdHR, RMSSD, pNN50, NN50, TINN, HF power and Total power of HRV parameters were not considered statistically significant. As observed in Figure 3 and Table 4, all eight top-ranked variables were significant in terms of *p*-value. Ultimately, these eight variables were chosen for model building and analysis.

**Table 4:** Measurements of HRV parameters and clinical signs of recruited patients

	**No MACE within 72 h (N = 673)**	**MACE within 72 h (N = 29)**	***p***
Clinical signs			
Glasgow Coma Scale	15 (15 to 15)	15 (15 to 15)	0.001
Temperature (°C)	36.4 (0.6)	36.4 (0.5)	0.871
Pulse rate (beats/minute)	79 (17)	86 (15)	0.008
Respiratory rate (breaths/minute)	18 (3)	20 (5)	0.010
Systolic BP (mmHg)	142 (28)	124 (31)	0.001
Diastolic BP (mmHg)	77 (15)	67 (17)	0.001
Oxygen saturation (%)	98 (4)	97 (4)	0.469
Pain score	2 (0 to 4)	3 (0 to 5)	0.358
HRV parameters			
aRR (s)	0.831 (0.171)	0.723 (0.139)	0.001
STD (s)	0.038 (0.028)	0.034 (0.020)	0.657
avHR (beats/minute)	75.545 (15.862)	86.122 (15.927)	0.001
sdHR (beats/minute)	3.618 (2.735)	4.328 (2.886)	0.099
RMSSD (s)	0.037 (0.039)	0.039 (0.307)	0.376
pNN50 (%)	7.294 (12.617)	7.978 (9.695)	0.198
NN50 (count)	23 (41)	31 (44)	0.220
Triangular index	3.009 (1.233)	2.481 (0.969)	0.025
TINN	0.134 (0.086)	0.105 (0.069)	0.086
LF power (ms^2^)	0.128 (0.074)	0.106 (0.092)	0.024
HF power (ms^2^)	0.125 (0.075)	0.139 (0.080)	0.303
Total power (ms^2^)	0.489 (0.110)	0.434 (0.177)	0.054
LF power norm (n.u.)	51.173 (20.535)	40.947 (22.966)	0.020
HF power norm (n.u.)	48.827 (20.535)	59.053 (22.996)	0.020
LF/HF	1.641 (1.869)	1.018 (0.910)	0.021

Data are shown as mean (standard deviation) or median (interquartile range, 25^th^ to 75^th^ percentiles). The *p*-values are calculated from the Mann–Whitney test.

MACE: major adverse cardiac events; For HRV parameters, refer to Table 1.

**Figure 4:**
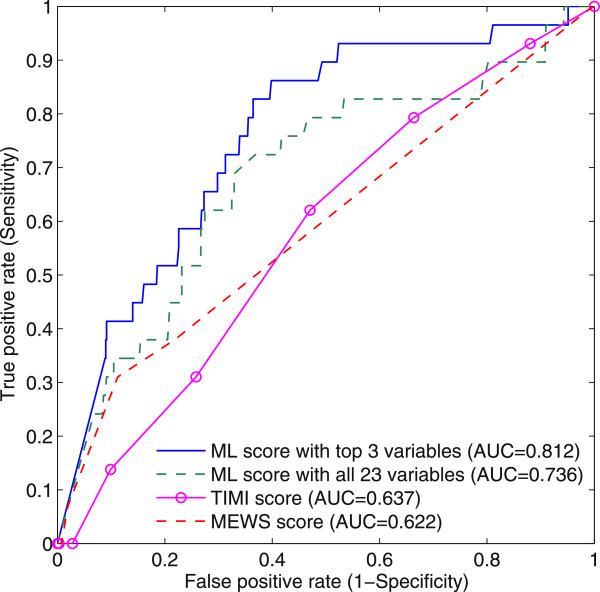
ROC curves of machine learning scores, TIMI and MEWS scores in predicting MACE within 72 h.

The performance of the intelligent scoring system with different numbers of selected variables is summarized in Table 5. The order of variables was determined from Figure 3 where each variable received its ranking with the proposed variable selection algorithm as shown in Figure 1. The combination of these variables therefore may not reflect any clinical meanings. The ML score with the top three selected variables was able to achieve the highest AUC among all other variable combinations. Figure 4 illustrates ROC curves produced by the ML score with the top three variables and the ML score with all 23 variables, TIMI score and MEWS score. A cut-off score was determined by the point that was nearest to the upper-left corner of the ROC curve. The ML score with top three variables produced an AUC of 0.812 (95% CI: 0.716 - 0.908) and a cutoff score of 43 gave a sensitivity of 82.8% (95% CI: 69.0% - 96.5%) and specificity of 63.4% (95% CI: 59.8% - 67.0%), while the ML score with all 23 variables had an AUC of 0.736 (95% CI: 0.630 - 0.841) and a cutoff score of 49 gave a sensitivity of 72.4% (95% CI: 56.1% - 88.7%) and specificity of 63.0% (95% CI: 59.3% - 66.6%). The TIMI score and the MEWS score achieved AUC values of 0.637 (95% CI: 0.526 - 0.747) and 0.622 (95% CI: 0.511 - 0.733), respectively.

**Table 5:** Top selected variables and their prediction performance

**Selected variables for model building**	**AUC**	**AUC 95% CI**
SBP	0.663	0.553 - 0.773
SBP, avHR	0.759	0.656 - 0.862
SBP, avHR, aRR	0.812	0.716 - 0.908
SBP, avHR, aRR, DBP	0.773	0.671 - 0.874
SBP, avHR, aRR, DBP, TI	0.763	0.660 - 0.865
SBP, avHR, aRR, DBP, TI, LF/HF	0.774	0.672 - 0.875
SBP, avHR, aRR, DBP, TI, LF/HF, HF norm	0.767	0.664 - 0.869
SBP, avHR, aRR, DBP, TI, LF/HF, HF norm, LF norm	0.768	0.666 - 0.870

Furthermore, a linear logistic regression model with forward selection was created where the predicted score ranges from 0 to 1. Six variables including Glasgow Coma Scale, respiratory rate, DBP, pain score, STD, and avHR were chosen for model building. The regression score yielded an AUC of 0.632 (95% CI: 0.564 - 0.7). A cutoff score of 0.11 gave a sensitivity of 58.0% (95% CI: 47.3% - 68.8%) and specificity of 55.7% (95% CI: 51.8% - 59.6%), respectively.

A pair-wise discrimination comparison of models is presented in Table 6 describing the outcomes among ML scores with and without variable selection, TIMI score and MEWS score. From the pair-wise comparison, it is observed that ML score with variable selection is correlated to ML score without variable selection (AUC diff. 0.076; *p*-value 0.28), and performed better compared to TIMI score (AUC diff. 0.175; *p*-value 0.005) and MEWS score (AUC diff. 0.190; *p*-value 0.011).

**Table 6:** Pair-wise discrimination comparison of AUC values of different risk prediction models

	**ML (Top 3)**		**ML (All 23)**		**TIMI**		**MEWS**	
**Method**	**Diff.**	***p***	**Diff.**	***p***	**Diff.**	***p***	**Diff.**	***p***
ML (Top 3)	-	-	-	-	-	-	-	-
ML (All 23)	0.076	0.280	-	-	-	-	-	-
TIMI	0.175	0.005	0.099	0.143	-	-	-	-
MEWS	0.190	0.011	0.114	0.213	0.015	0.809	-	-

Diff: AUC difference; *p*: the *p*-value of AUC difference between two models.

## Discussion

This study is a continuation of our previous work on applying both HRV and clinical signs for outcome prediction [10]. In this study, we investigated the use of variable selection to choose the most relevant predictors of MACE within 72 h. The ML method was able to more accurately identify patients who met the primary outcome than MEWS and TIMI scores. Only 3 variables including systolic BP, HRV parameters avHR and aRR were required to achieve the best risk prediction performance. While 5-min ECG is not the standard of care, we have demonstrated its feasibility and effectiveness of risk prediction. The analysis of short recordings of ECG HRV has potential to benefit triage in the ED, where timely response is essential. Furthermore, only SBP instead of all eight clinical signs is required for risk prediction, which dramatically simplifies the scoring system and saves a lot of time in measurement.

Many scoring systems have been proposed to measure the risk of adverse events in acute patients. However, no study has yet been done on performance-driven model building with random forest (RF)-based variable selection. With the RF technique, we derived a novel variable selection process integrating the strengths of both machine learning and statistics. Artificial intelligence has been used in the medical field for many years [16,33]. Due to the fact that positive samples (patients with MACE) are a small proportion of the dataset, common machine learning-based model building usually fails to achieve effective risk analysis. The trained model tends to over-fit due to the predominance of negative samples (patients without MACE). Techniques for handling imbalanced data has been well summarized in He and Garcia [25]. Three major techniques are widely used, namely: sampling methods, cost-sensitive methods, and kernel-based and active learning methods. Each type of method has its pros and cons. Because of its simple yet effective structure, the sampling method was adopted together with random forest to create a novel variable selection algorithm.

A total of 23 variables consisting of 15 HRV and eight clinical signs were investigated for their associations with the primary outcome. The results showed that several HRV parameters and clinical signs were significant in predicting MACE within 72 h. It has also been reported that patients with “normal” vital signs may be more ill than they appeared [34], and therefore predictors other than traditional vital signs are needed to increase the chance of detecting critically ill patients. We have previously discovered that a combination of HRV and clinical signs outperformed HRV alone and clinical signs alone [17,24]. It is shown in this study that a few significant predictors were able to improve performance as well as to shorten decision making time. Both RF-based and statistics-based (by means of *p*-value) variable selection methods were able to pick up significant variables such as SBP, avHR and aRR. It is worth noting that avHR (average of the instantaneous heart rate) and aRR (average width of the RR interval) were correlated and both were selected for building the scoring model. In a conventional statistics approach, these two variables will not appear in the same prediction model. However, in a machine learning method, variable selection is performance-driven where both prediction performance and statistical significance play important roles in the selection process.

With these selected variables, triage (in terms of risk prediction) can be done within a shorter time to determine priority of patients’ treatments. Fast response is of great value in triage because resources are usually limited for all patients to receive immediate attention. In this scenario, a few yet significant variables will benefit the triage from two aspects. From software modeling perspective, they will maintain a balance between model complexity and prediction performance because including too many predictors may lead to a loss in precision, whereas omitting important variables may result in biased prediction [23]. From hardware design perspective, fewer variables that require lesser time and effort to obtain will make a triage device simpler and faster in real-time usage.

Our study has several limitations. Since this is a single-center pilot study at an acute tertiary hospital in Singapore, the number of recruited patients is small and our findings may not be generalizable to other populations. The endpoints in this study are heterogeneous while the event rate is still low. This creates difficulty in associating predictive variables with specific outcomes. The TIMI outcomes (death, AMI, and revascularization within 30 days) might be adopted to standardize model derivation and for comparison across several risk scoring methods in chest pain patients presented to the ED.

While we adopt random forest for variable selection, there are several other techniques available for selecting significant variables [18]. Small sample size may lead to overestimation even though we have adopted the leave-one-out cross-validation scheme. The ideal way is to validate the method on a separate dataset. Given more available data in the future, we will be able to derive a model from one dataset and validate its performance on another. Finally, in variable selection we created the subsets by using 1:1 ratio between MACE and no MACE patient groups, which may potentially lead to bias.

In future work, exploration of other potential predictive variables will be considered. Gender has been reported to be useful for patient outcome prediction [35]; 12-lead ECG changes and bedside qualitative cardiac troponin [36] could also be added to the algorithm to enhance prediction performance. Also, including demographic and medical history variables in the predictive model may be useful. In this study, eight top-ranked variables were chosen where the number was empirically determined to create a trade-off between accuracy and efficiency. Therefore, finding a method to decide the optimal number of variables is necessary so that the proposed variable selection method can be generalized in other applications.

## Conclusions

This study expands our understanding that only a few predictors are needed in risk stratification of MACE within 72 h. With the proposed variable selection method, a machine learning scoring system outperforms traditional risk stratification systems such as TIMI and MEWS. We have seen that blood pressure and some HRV parameters achieved better prediction performance than a larger set of clinical signs and HRV parameters. It shows that more predictors do not necessarily guarantee better prediction results. Furthermore, variable selection presents potential to simplify scoring systems with just a few relevant measures as predictors, and the machine learning based scoring system demonstrates its adaptability to changes of variables.

## Abbreviations

ACS: Acute coronary syndrome; AMI: Acute myocardial infarction; AUC: Area under ROC curve; BP: Blood pressure; CABG: Coronary artery bypass graft; CIRB: Centralized Institutional Review Board; DBP: Diastolic BP; ECG: Electrocardiogram; ED: Emergency department; GCS: Glasgow coma scale; GRACE: Global registry of acute coronary events; HF: High frequency; HR: Heart rate; HRV: Heart rate variability; IABP: Intra-aortic balloon pump; LF: Low frequency; LOOCV: Leave-one-out cross-validation; MACE: Major adverse cardiac events; MEWS: Modified early warning score; ML: Machine learning; PCI: Percutaneous coronary intervention; RF: Random forests; ROC: Receiver operating characteristic; SBP: Systolic BP; SGH: Singapore general hospital; SingHealth: Singapore health services; SpO_2_: Oxygen saturation; SVM: Support vector machine; TI: Triangular index; TIMI: Thrombolysis in myocardial infarction; VLF: Very low frequency; VT: Ventricular tachycardia.

## Competing interests

Marcus Eng Hock Ong and Nan Liu have a patent filing related to this study (System and method of determining a risk score for triage, Application Number: US 13/791,764). Marcus Eng Hock Ong and Zhiping Lin have a patent filing related to this study (Method of predicting acute cardiopulmonary events and survivability of a patient, Application Number: US 13/047,348). Marcus Eng Hock Ong and Zhiping Lin also have a licensing agreement with ZOLL Medical Corporation for the patented technology. There are no further patents, products in development or marketed products to declare. All the other authors do not have either commercial or personal associations or any sources of support that might pose a conflict of interest in the subject matter or materials discussed in this manuscript.

## Authors’ contributions

NL and MEHO planned and established the project, performed data analysis, and drafted the manuscript. ZXK, JG, and BPT performed data collection and data analysis. BH performed detailed statistical analysis. ZL drafted the manuscript and reviewed critical revisions. All authors took part in manuscript writing and approved the final manuscript.

## Pre-publication history

The pre-publication history for this paper can be accessed here:

http://www.biomedcentral.com/1472-6947/14/75/prepub
